# A Comprehensive View of the β-Arrestinome

**DOI:** 10.3389/fendo.2017.00032

**Published:** 2017-03-06

**Authors:** Pascale Crépieux, Anne Poupon, Nathalie Langonné-Gallay, Eric Reiter, Javier Delgado, Martin H. Schaefer, Thomas Bourquard, Luis Serrano, Christina Kiel

**Affiliations:** ^1^INRA, UMR85, Unité Physiologie de la Reproduction et des Comportements, Nouzilly, France; ^2^«Biology and Bioinformatics of Signaling Systems (BIOS)» Group, CNRS, UMR7247, Nouzilly, France; ^3^Université François Rabelais, Tours, France; ^4^IFCE, Nouzilly, France; ^5^EMBL/CRG Systems Biology Research Unit, Centre for Genomic Regulation (CRG), Barcelona Institute of Science and Technology, Barcelona, Spain; ^6^Universitat Pompeu Fabra (UPF), Barcelona, Spain; ^7^Institució Catalana de Recerca i Estudis Avançats, Barcelona, Spain

**Keywords:** hub proteins, β-arrestins, G protein-coupled receptors, protein/protein interaction network, systems biology

## Abstract

G protein-coupled receptors (GPCRs) are membrane receptors critically involved in sensing the environment and orchestrating physiological processes. As such, they transduce extracellular signals such as hormone, neurotransmitters, ions, and light into an integrated cell response. The intracellular trafficking, internalization, and signaling ability of ligand-activated GPCRs are controlled by arrestins, adaptor proteins that they interact with upon ligand binding. β-arrestins 1 and 2 in particular are now considered as hub proteins assembling multiprotein complexes to regulate receptor fate and transduce diversified cell responses. While more than 400 β-arrestin interaction partners have been identified so far, much remains to be learnt on how discrimination between so many binding partners is accomplished. Here, we gathered the interacting partners of β-arrestins through database mining and manual curation of the literature to map the β-arrestin interactome (β-arrestinome). We discussed several parameters that determine compatible (AND) or mutually exclusive (XOR) binding of β-arrestin interactors, such as structural constraints, intracellular abundance, or binding affinity.

## Introduction

G protein-coupled receptors (GPCRs) are the largest class of integral membrane receptors involved in signal transduction from the cell environment inward. Their cognate ligands encompass a vast array of structural entities, including glycoprotein hormones, chemokines, peptide neurotransmitters, ions, as well as sensory molecules such as light, odorants, or taste ligands. Given this plethora of ligands, GPCRs are involved in many physiological and pathological processes, making them prime target classes for drug discovery. As major transducers of GPCR activation, β-arrestins 1 and 2 (aka arrestins 2 and 3) represent a particular subtype of hubs, their conformation and activation being dependent upon their association to ligand-bound receptor. β-arrestins contribute to connect the extracellular *milieu* to the intracellular space, by desensitizing and internalizing the receptor in order to avoid endless second messenger production at the plasma membrane, and by scaffolding signaling modules that can be activated independently, or in conjunction with G proteins. A key factor in determining β-arrestin binding specificity is their sensitivity to the phosphorylation barcode of the receptor, which dictates the affinity of the interaction and the conformation they adopt (1, 2). In particular, some agonist-stimulated GPCRs are phosphorylated on distinct sites by G protein-coupled receptor kinases (GRK) 5 or 6, and by GRK2 or 3 (3–7). These combinatorial phosphorylations impart variable conformations of the β-arrestins recruited at the GPCR carboxy terminus. Consequently, β-arrestins recruited on the receptor at GRK5 and 6 phosphorylated sites lead to the assembly of a signalosome, such as the ERK MAP kinase module, while β-arrestins recruited at GRK2 and 3 phosphosites promote receptor internalization (1, 2, 7–10). In addition, β-arrestins binding at the GPCR carboxy terminus can co-exist with G protein binding in endosomes, which sustains G protein signaling inside the cell (11). Finally, some interactors also bind free β-arrestins, such as microtubules, calmodulin, and the E3 ubiquitin ligases MDM2 and Parkin (12) among others, extending the role of β-arrestins to GPCR-independent signaling.

Although more than 400 of their protein partners have been identified (13), the relatively small size (45 kDa) of β-arrestins and their limited potential interaction interface, estimated as 17,000 Å^2^, precludes their interaction with as many interacting partners at a time. By analogy with Boolean logic gate operators of electronic circuits, compatible surface interactions can be distinguished from mutually exclusive interactions with the “AND” and “XOR” operators, respectively (14–16). A prominent cause of XOR interactions relies on structural constraints imposed by the availability of β-arrestin docking sites, as illustrated by the interaction between β-arrestin 2 and tubulin, Ca^2+^-dependent calmodulin, and GPCR, which all use the same binding site (17). Protein abundances together with affinities may also invoke competition between binding partners for a common docking site on β-arrestins and ultimately contribute to cell- and tissue-specific signaling responses. Here, we gathered the current knowledge on interaction partners for β-arrestins 1 and 2 (encoded by the *ARRB1* and *ARRB2* genes, respectively) to provide a comprehensive map of the “β-arrestinome.”

## The β-Arrestinome

In order to retrieve β-arrestin protein partners and reconstruct a comprehensive β-arrestin interaction map, we searched for β-arrestin-binding partners in the literature and in publicly available protein interaction databases. First, most of the interactions were extracted from a previously published proteomics analysis of the β-arrestin interactome, following coimmunoprecipitation of FLAG-tagged β-arrestins 1 and 2 in HEK293 cells stimulated by angiotensin II, and peptide identification by mass spectrometry (MS) (MudPIT and LC–MS/MS) (13). Then, some more β-arrestin partners were sequentially retrieved from queries in NetPath (release 9) (18), BioGRID (3.4 version) (19), Mentha (25-09-2016 release) (20), and HIPPIE (v2.0 24-06-2016) (21) databases. All the relevant experiments were verified in the original publications. Finally, the analysis was completed by manual curation of the literature. All this information was used to build the β-arrestin interactome. To get all interactions between β-arrestin partners, the interaction networks were inferred in HIPPIE (21) that automatically converts protein–protein interactions into a connected network.

**Figure 1:**
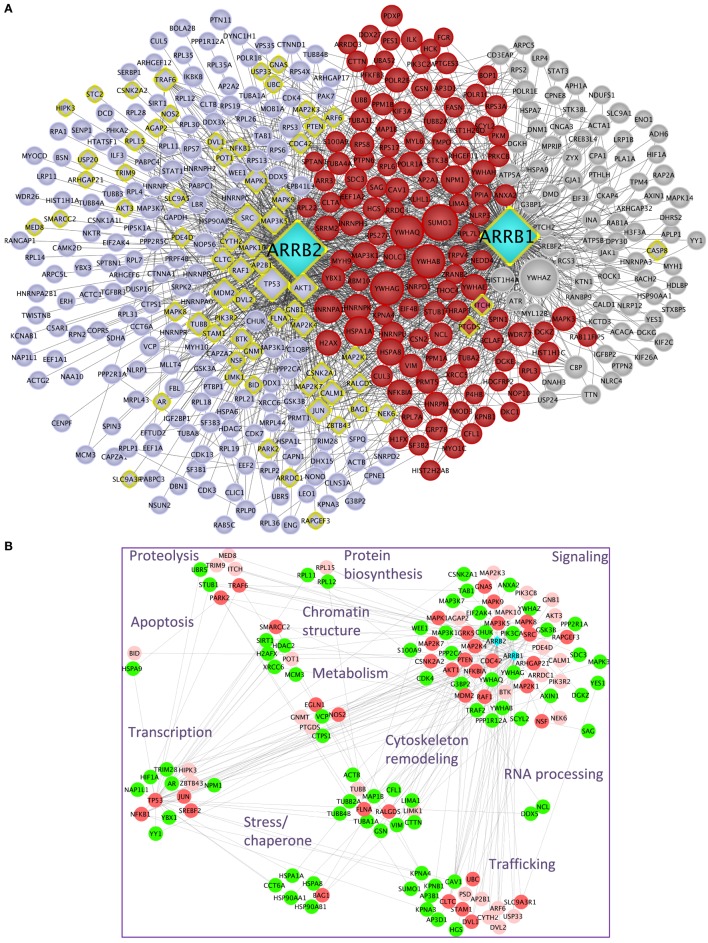
**(A)** β-Arrestins 1 and 2 interactome, represented as a non-directional interaction graph in Cytoscape, according to Table S1 in Supplementary Material. Each node represents a protein, whereas each edge represents an interaction, as reconstituted with the HIPPIE algorithm to visualize not only interactions with β-arrestins but also interactions among β-arrestin partners. Interactions experimentally shown for one or the other β-arrestin selectively are in gray for β-arrestin 1 and blue for β-arrestin 2, whereas the interactions demonstrated for both are in red. β-arrestins are in turquoise. Yellow diamonds indicate partners interacting directly at least with one β-arrestin. The size of each node is proportional to its degree of connectivity. **(B)** Projection of the first neighbors (*n* + 1, green) of the experimentally validated direct interaction partners of β-arrestins (*n*, vermilion) on the whole β-arrestinome, as determined by IPA. The direct interaction partners for which no first neighbors have been identified within the β-arrestinome are in light pink. The proteins have been sorted according to their biological function. For simplification purpose, the receptor class has been omitted. Symbols indicated are the Uniprot gene names (http://www.uniprot.org).

Upon public database queries and manual curation of the literature, 282 experimentally validated interactions were recovered for β-arrestin 1 and 374 for β-arrestin 2 (Table 1). The whole β-arrestinome and interactions among partners, visualized using Cytoscape (22), comprises 429 unique nodes and 1,599 unique edges (Figure 1A). We discriminated direct (yellow diamonds) and indirect interactions. Direct interactions have been revealed by yeast two-hybrid or by *in vitro*-reconstituted complex of purified recombinant proteins (called “direct” in Table S1 in Supplementary Material). Interactions uncovered in the same macromolecular complexes by immunoprecipitation or pull down assays (called “undetermined” in Table S1 in Supplementary Material) could be direct or not. From this view, the partners common to both β-arrestins (highlighted in red) are involved in some of β-arrestin key biological functions, such as intracellular trafficking (SRC, MDM2, and CAV1), cell signaling (MAPK pathways, 14-3-3 proteins, SRC, AKT1, and CALM1), gene transcription (PRMT5, NPM1, and POLR2E), or cytoskeleton remodeling (CDC42, LIMK1, FLNA, RALGDS, and CFL1). Others are involved in RNA processing, protein biosynthesis, and chromatin remodeling. The data that impose the heaviest weight on our whole analysis mainly rely on a single study using a common anti-FLAG antibody to immunoprecipitate both β-arrestins, prior to two distinct state-of-the-art MS analyses (13), which likely limits random under-representation of the partners of one or the other β-arrestin.

**Table 1:** **Number of β-arrestins 1 and 2 partners retrieved from manual curation of the literature and by queries in publicly available databases**.

Query^a^	β-Arrestin 1	β-Arrestin 2
Xiao et al. (13)	158	244
NetPath	21	27
BioGRID	40	27
Mentha	10	7
HIPPIE	4	30
Manual curation	49	39
Total	282	374

*^a^In each subsequent source, the additional interactions found with respect to the previous one were sequentially added*.

It is conceivable that hub proteins, such as TP53, MDM2, SUMO1, and 14-3-3 proteins support many indirect interactions between β-arrestins and their partners. In addition, since β-arrestins could heterodimerize (23), any of the two β-arrestins could indirectly connect the other one with its own binding proteins.

In light of the present knowledge, about 17.2% of all the reported partners of both β-arrestins, not including the receptors, have been experimentally validated as direct binders [57 (22.7% of the direct interactors) for β-arrestin 1 and 44 (13.1%) for β-arrestin 2, the remaining ones binding to both β-arrestins] (Table S1 in Supplementary Material). In total, 26 interactions demonstrated as direct ones are common to both β-arrestins, representing 25.7% of the direct ones and 4.4% of the total. Besides these experimentally validated direct interactions, the question remains open as to whether the other interactions in the whole β-arrestinome also include direct ones.

Considering a subnetwork composed of β-arrestin direct interactors denoted as *n*, we artificially extended it with the respective first neighbors of these nodes (*n* + 1). Then, these *n* + 1 nodes were projected on the whole β-arrestinome (Figure 1B). By these means, 112 (33%) of nodes coincided with these *n* + 1 interactions, when no filter was applied on the type of interaction, i.e., both functional and physical interactions were considered. When considering only protein–protein interactions, this ratio still reaches 21% (24% when adding phosphorylation relationships and 25% when considering ubiquitination). Since *n* + 1 nodes are in close contact with β-arrestin direct binders, these nodes have to be considered as putative direct interactors of β-arrestins also. For example, PTEN has previously been shown to activate the actin depolymerization factor cofilin (24), and we propose that β-arrestins could scaffold this complex, although cofilin has not been shown as a β-arrestin direct binder yet. This possibility deserves to be experimentally addressed in the future.

## Structural Mapping of β-Arrestin Direct Interactors

It was proposed earlier that extensive conformational flexibility of β-arrestins allows them to adopt many different interfaces, which may provide an explanation for why their relatively small surface can accommodate so many different binding partners. In an attempt to support this view, we used PepX, an algorithm that has been demonstrated earlier to predict docking sites with high precision based on a library of peptide–peptide interactions (25). Using different β-arrestin protein structures as the input (PDB entries 2WTR, 4JQI, and 3P2D), PepX was used to determine the accessible conformational space for possible bound peptide backbone fragments through interaction constraints using them as anchors. The algorithm is based on the finding that protein–peptide binding occurs for a certain limited number of conformations. Thus, a heuristic algorithm (CSP) is applied in order to reduce the conformational search space of all overlapping fragments of the PepX database (containing more than 7 × 10^6^ interactions from 1,431 PDB structures representing the structural coverage of protein–peptide interactions). This is followed by an unsupervised clustering method to predict possible binding sites and peptide conformations. In the first step, this algorithm predicts all compatible backbone conformations (“fitting”). In the second and more time-consuming step, the interface can be refined by taking side chain modeling into account (“binding”).

**Figure 2:**
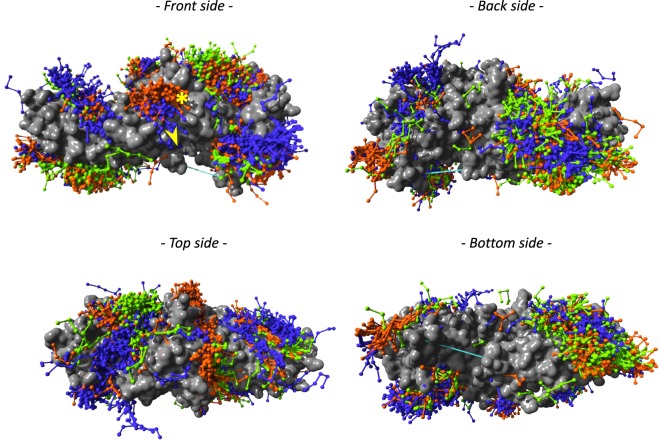
**PepX docking predictions with peptide length 5 has been done on three template structures, but only the surface representation of β-arrestin 2 (PDB entry 3P2D) is shown here, in gray**. Peptides are colored according to the template structure that was used for PepX predictions: β-arrestin 1 (PDB entry 2WTR) in orange, β-arrestin 2 (PDB entry 3PD2) in blue, and β-arrestin 1 in active conformation (PDB entry 4JQI) in green. The yellow star shows the PDE4D- and GNAS-binding site; the yellow arrow points to the RAF1-binding site.

Using three template structures, namely, dimeric bovine β-arrestin 1 (unpublished), active rat β-arrestin 1 bound to a V2 receptor phosphopeptide (1), and bovine β-arrestin 2 (26), we independently predicted the compatible backbone conformations using different peptide lengths (lengths 5, 6, 7, 8, and 9). With peptide length 5, several regions of β-arrestin surface were covered, which were proposed experimentally as docking sites (Figure 2; Table S2 in Supplementary Material). Most peptides were predicted to bind to overlapping sites on both β-arrestins 1 (orange) and 2 (green). In contrast, in several instances, the peptide clouds on active (blue) *versus* inactive (orange) β-arrestin 1 did not entirely match. For example, as viewed on the front side view, several peptides appeared to bind to active β-arrestin 1 in a region located in the vicinity of L33 where it interacts with PDE4D or GNAS (yellow star) (Table S2 in Supplementary Material), whereas a distinct peptide cloud was predicted to interact with inactive β-arrestin 1, in the region where RAF1 interacts (yellow arrow).

The larger the surface that was covered by peptides with compatible backbone conformations, the more template structures were used. This supports the idea that, depending on the activation state of β-arrestins, even small structural changes, as observed in different X-ray structures, can provide different interfaces for potential β-arrestin interacting partners.

## XOR/AND Interactions in the β-Arrestinome in Light of Expression Levels and Affinities

**Figure 3:**
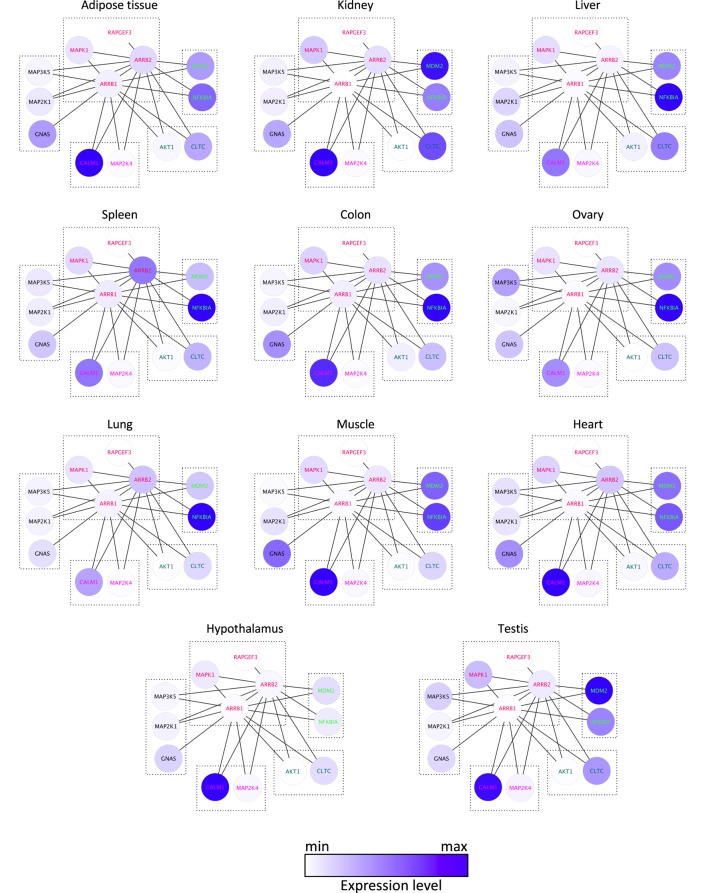
**Comparison of the mRNA expression level of 13 direct binding partners of β-arrestins 1 and 2 in 11 human tissues, as indicated, according to Table S3 in Supplementary Material**. XOR interactions are boxed, according to the docking site identified experimentally (see Table S2 in Supplementary Material).

Common XOR docking sites on β-arrestins do not necessarily lead to competition for binding among interaction partners. Clearly, the relative abundance of the common hub (here β-arrestin) and the partners are important to consider, because they govern the equilibrium of complex formation within the cell. To analyze how protein expression levels relate between β-arrestin partners, quantitative information for β-arrestins 1 and 2 and 82 direct interactors including receptors was retrieved from RNAseq datasets of gene expression levels in 11 human normal tissues (http://www.medicalgenomics.org/) (Table S3 in Supplementary Material). The relative expression levels of direct β-arrestin partners were represented with the continuous mapping representation of Cytoscape. β-arrestin 1 mRNA appeared less abundant than β-arrestin 2 mRNA, although analysis of the respective protein levels shows the opposite (14, 27), suggesting posttranscriptional mechanisms to enhance β-arrestin 1 intracellular protein level. Importantly, the cumulated amount of all 80 direct interaction partners greatly over-exceeds the abundance of the two β-arrestins. This suggests that in those cases where interaction partners bind to a common docking site, competition between partners will exist. In this case, the relative abundance of each partner may be decisive (Figure 3; Table S3 in Supplementary Material). As represented with the Cytoscape software (22), both NFKBIA and MDM2 interact with the N terminus of β-arrestins (1–60), and their relative expression level in human tissues suggests that the binding equilibrium might be displaced in favor of MDM2 in kidney and testes, whereas NFKBIA is much more abundant in other tissues, except in adipose tissue and heart where both genes are equally expressed. Likewise, GNAS, MAP2K1, and MAP3K5 are predicted to compete for the same docking site on β-arrestins on amino acids in the vicinity of the β-arrestin polar core, and their respective expression level varies among tissues, e.g., in kidney and in ovary.

Differences in the affinity of several proteins suspected to compete for identical docking site on β-arrestins may also affect apparent competitive interactions. For example, tubulin and calmodulin bind β-arrestins with a *K*_D_ in the micromolar range, to be compared with the interaction with activated GPCR, which is in the nanomolar range (17). Given the affinity constant of the respective proteins, the receptor would win the race in a context of limiting quantities of β-arrestins. Such conditions are encountered for class A GPCR that interact transiently with β-arrestin 2, for which the affinity for β-arrestin 2 is thought to be higher than for β-arrestin 1, and that are recycled back to the plasma membrane shortly after internalization (28). As mentioned above, at the protein level, β-arrestin 2 is generally expressed at much lower level than β-arrestin 1 in tissues; hence, this imbalance may have profound consequences on the duration of GPCR intracellular trafficking. However, variations in the concentration of the various proteins are expected to compensate for differential affinities, following the law-of-mass action.

**Figure 4:**
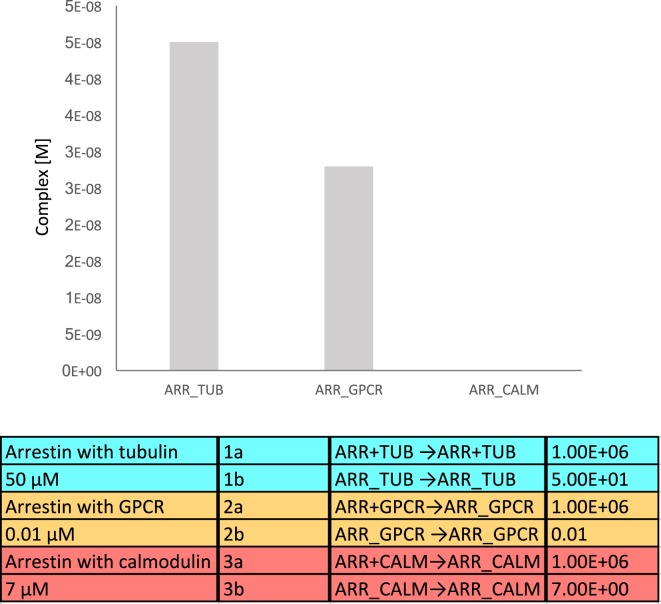
**Equilibrium binding model of β-arrestin interacting with tubulin, G protein-coupled receptor (GPCR), and calmodulin**. The equilibrium complex concentrations are depicted in the bar diagram.

We generated a simple mathematical model that includes binding affinities and average endogenous cellular concentrations, where tubulin, the GPCR, and calmodulin compete for binding to β-arrestins. The affinity (*K*_D_) for β-arrestin 1 with tubulin was assumed to be 50 μM, the affinity of β-arrestins with a GPCR as 10 nM, and the affinity of β-arrestin 1 with calmodulin as 7 μM (29). To translate affinities into kon and koff values, we used similar kon values (10^6^ M^−1^ s^−1^) and calculated koff from the relation, *K*_D_ = koff/kon. The volume of a mammalian cell was assumed to be 2.34 × 10^−15^ L (source: HeLa cells in Bionumbers). Endogenous tubulin concentration was estimated as 4 × 10^−4^ M (30), and endogenous calmodulin concentration as 8 × 10^−9^ M (27). Concentrations of β-arrestin and GPCR were estimated as 8 × 10^−8^ M (27). The equilibrium binding model was generated using the Intrinsic Noise Analyzer simulation software (31), followed by a time-course analysis until equilibrium. With this model, we found that approximately half of the available β-arrestins was bound to tubulin (Figure 4). As tubulin is so highly expressed in cells, an interesting consideration is whether binding of tubulin is specific or just results from the fact that it has a poly-Glu C-terminal tail, which may mimic the phosphorylated GPCR.

Yet another possibility is that the receptors, calmodulin and tubulin, interact with different subpopulations of β-arrestins, with different biological outcomes. For example, the ERK MAP kinase module that is able to interact with β-arrestins at the GPCR can also be sequestered by β-arrestins on the microtubules, which blunts ERK activity (32).

Noteworthy, the conformation of β-arrestins themselves, depending on their binding to the receptor, is also a factor that crucially affects the affinity for additional interactions, as recently demonstrated for β-arrestin 1 binding to clathrin (2, 33, 34).

## Two Examples of Mechanistic Maps within the β-Arrestinome

**Figure 5:**
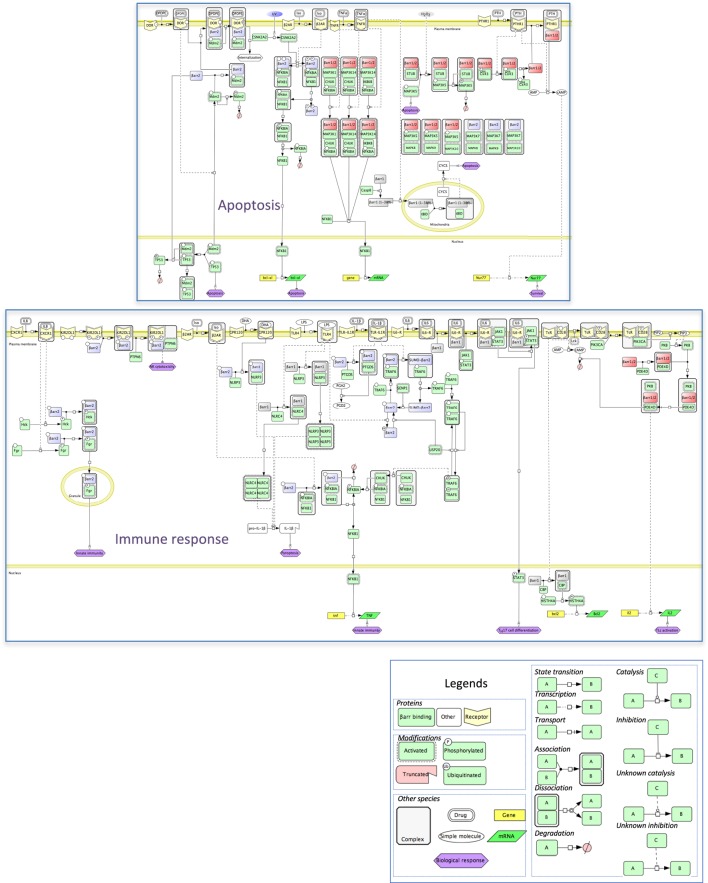
**Mechanistic map of β-arrestin dependence of two intertwined functional cell responses, apoptosis, and immune response**. Biochemical reactions were edited in the Cell Designer format (see included legend). The molecular species not included in the β-arrestinome are in white, membrane receptors are in yellow, β-arrestins 1 or 2, and both β-arrestins are colored as above. Both cell responses connect at the level of the NFKB pathway (data not shown).

In an attempt to provide more mechanistic insight onto the integration of these numerous protein–protein interactions with β-arrestins, a molecular interaction map was inferred and represented in standardized graphical format, using the Cell Designer interface (35), version 4.4. This map recapitulates the relationships between β-arrestins and their binding partners in two physiological responses in which β-arrestins are involved. That is, β-arrestins exquisitely fine-tune the balance between pro- and anti-apoptotic signals in different cell types in general and in the immune system in particular. These mechanisms exhibit prominent physiological relevance, and their disturbance causes severe pathologies, such as autoimmunity or septic shock. The signal demonstration for the physiological role of arrestins in cell death/survival was that visual arrestin impairment accelerates retinal degeneration (36). At the molecular level (Figure 5), β-arrestins inhibit NFKB anti-apoptotic activity by stabilizing the NFKBIA inhibitor. How this is achieved is not entirely clear because, although β-arrestins scaffold the inhibitory upstream kinases of NFKBIA, such as CHUK or IKBK, together with NFKB and NFKBIA (37), they do not seem to alter IKBK enzymatic activity (38). Notably, β-arrestin-mediated NFKB inhibition is sensitive to agonist binding on the muscarinic M1, AT2R (37), or β2-AR receptors (38). For example, by preventing β-arrestin 2 phosphorylation on S361 and S383 by casein kinase 2, isoproterenol stimulation counteracts the anti-apoptotic role of NFKB in cells exposed to UV irradiation (39). GPCR activation may also lead to the formation of a receptor/β-arrestin 2/MDM2 complex, with two major consequences: first, β-arrestin ubiquitination by MDM2 enhances receptor internalization (40); second, the interaction between β-arrestin and MDM2 pumps the latter out of the nucleus, which limits TP53 degradation and promotes its pro-apoptotic function (41). Regulation of the availability of β-arrestins themselves conditions the outcome on cell survival, as shown with Cx43-mediated β-arrestin sequestration in osteoblasts (42). More directly, caspase also triggers β-arrestins to interact with mitochondrial executioners of apoptosis, such as t-BID (43). However, β-arrestin anti-apoptotic functions have also been demonstrated in oxidative stress conditions, by triggering MAP3K5 (ASK1) proteosomal degradation (44), or by counteracting FPR, V2R, CXCR2, or AT1R pro-apoptotic action on mitochondrial caspases (45).

Apoptosis is a programmed process that shapes the development as well as homeostasis of the immune system. β-Arrestins intervene in both adaptive and innate immunity (Figure 5). As shown in mouse models, β-arrestin 2 stimulates the activity of receptors that negatively regulate NK cell cytotoxicity, such as the KIR2DL1 receptor (46) or dampens the activity of pro-inflammatory receptors, again by preventing NFKBIA degradation and NFKB-induced cytokine gene expression, *via* interaction with TRAF6 (47). Likewise, ligand binding to GPCR, such as isoproterenol-stimulated β2-AR, may lead to anti-inflammatory outcomes (38). In addition, β-arrestins also positively regulate innate immunity, as shown *in vitro* by their ability to support the degranulation of polymorphonuclear neutrophils, through complexation with Src-related tyrosine kinases (48). In the specific context of inflammation, both β-arrestins seem to play opposite roles, with β-arrestin 1 favoring the oligomerization of NLRP3 and NLRC4 inflammasomes, in bone marrow-derived macrophages (49), whereas in these cells, β-arrestin 2 is involved in the DHA-mediated inhibition of the NLRP3 or NLRP1 inflammasomes through GPR120 and GPR40, two receptors of long-chain fatty acids (50).

Homeostasis of CD4^+^ Lc, the cellular support of adaptive immunity, is also regulated in part by β-arrestin 1 that favors their survival by enhancing *BCL2* expression level in these cells, through epigenetic modifications of H4 histone (51). The fact that *ARRB1* knockout mice were much more resistant to experimentally induced autoimmune encephalomyelitis highlights a protective role against apoptosis, in CD4^+^ TLc.

## Concluding Remarks

Here, we propose a snapshot of what β-arrestin signaling networks could look like. However, how β-arrestins orchestrate GPCR-dependent as well as GPCR-independent signaling is rather related to a movie, with simultaneous molecular events occurring in different places, and changing over time. Hence, decisive advance in understanding β-arrestin cellular functions requires elaborating dynamic models taking into account the affinities of competitors in XOR interactions, protein relative abundance in tissues, and subcellular distribution of the respective complexes, with, on top of that, β-arrestin dimerization adding even more complexity. Although not trivial to measure routinely, as more and more *K*_D_ of interaction between signaling proteins become available, the dynamics of complex formation within the β-arrestinome will be understood more accurately.

A dynamic view of β-arrestin-dependent signaling networks may lead to more practical advances, because GPCR are the top class drug targets. β-arrestins are being intensively scrutinized in this respect because they transduce beneficial or detrimental effects; hence, biased agonists and modulators that select parts of a GPCR-induced signaling repertoire are being ardently sought after (52). As testified by the intricacy and complexity of the signaling networks that they regulate, it is of great interest to disrupt some but not all of their interactions. To confirm predicted steric hindrance, co-crystals of β-arrestins with their direct interaction partners need to be provided, although this is technically and scientifically challenging. In addition, many works in the recent past have attempted, and partly succeeded, in discriminating the regulatory mechanisms that govern β-arrestin-mediated desensitization *versus* signaling. Future experiments will hopefully permit discrimination between the GPCR-dependent signaling regulatory function of β-arrestins and their GPCR-independent contribution to cellular compartmentalization. By the same token, new therapeutic agents could be able to disrupt a particular β-arrestin interaction in one site of the cell but preserve its integrity in another place. Attaining this Rosetta stone would pave the way for the design of drugs that interfere with selective interactions among spatially restricted β-arrestin subpopulations.

## Author Contributions

PC and CK designed the study. PC, LS, NL-G and AP did the analysis from the literature. MS did the statistical analyses. AP, JD, and TB commented the 3D structures presented. PC, AP, ER, LS, and CK wrote the paper, with contributions of all the other authors.

## Conflict of Interest Statement

The authors declare that the research was conducted in the absence of any commercial or financial relationships that could be construed as a potential conflict of interest.
